# 10-year-long survival in a PD patient with severe calcifying encapsulating peritoneal sclerosis treated with tamoxifen: a case-report

**DOI:** 10.1186/s12882-020-01769-x

**Published:** 2020-03-31

**Authors:** Vassilios Liakopoulos, Panagiotis I. Georgianos, Vasilios Vaios, Stefanos Roumeliotis, Apostolos Karligkiotis, Pantelis E. Zebekakis

**Affiliations:** Peritoneal Dialysis Unit, 1st Department of Medicine, AHEPA Hospital, Aristotle University of Thessaloniki, St. Kyriakidi 1, GR54636, Thessaloniki, Greece

**Keywords:** Encapsulating peritoneal sclerosis, Peritoneal dialysis, Mortality, Tamoxifen

## Abstract

**Background:**

Encapsulating-peritoneal-sclerosis (EPS) is a rare, but serious and life-threatening complication of peritoneal dialysis (PD). Treatment of EPS consists of discontinuation of PD and maintenance of nutritional status, whereas the role of corticosteroids, tamoxifen and other immunosuppresive agents is not yet fully elucidated.

**Case-presentation:**

We report the case of a 28-year-old patient, who developed a severe form of calcifying EPS after a 6-year-long therapy with automated PD. The clinical presentation was severe with repeated episodes of total bowel obstruction, weight loss and malnutrition that mandated his prolonged hospitalization. Initial treatment included corticosteroids and tamoxifen (20 mg/day) with a clinically meaningful improvement in gastrointestinal function and nutritional status over the first 6–12 months. Corticosteroids were discontinued at 18 months, but owing to persistence of calcifying lesions and peritoneal thickening in repeated computed-tomography (CT) scans, tamoxifen remained unmodified at a low-dose of 20 mg/day for a 10-year-long period. During follow-up, the patient remained symptoms-free in an excellent clinical condition and the CT findings were unchanged.

**Conclusions:**

Long-term administration of tamoxifen was not accompanied by any drug-related adverse effects and potentially exerted a beneficial action on down-regulation of inflammatory and fibrotic processes and improvement of gastrointestinal function, nutritional status and overall health-related quality of life.

## Background

Encapsulating-peritoneal-sclerosis (EPS) is a rare, but devastating complication of peritoneal dialysis (PD) [1]. In registry studies its incidence is ranging from 0.9 to 3.5% [2–4], depending mainly on the duration of PD therapy of participants. Although the pathogenesis of EPS is not yet fully elucidated, several lines of evidence suggest that EPS reflects a progressive inflammatory and fibrotic process that results in excessive peritoneal thickening and encapsulation of the intestine [1, 5]. The spectrum of clinical manifestations is wide and often non-typical, a fact that raises particular difficulties in early detection of this serious complication. In severe cases, EPS is accompanied by a huge morbid burden, including total bowel obstruction, weight loss, malnutrition, infection and death [1]. The mortality hazard attributable to this complication is reported to be as high as 50% [3, 4, 6], particularly within the first year of diagnosis.

The prevention of EPS is an area surrounded by substantial controversy that is reflected by the absence of a clear consensus on the optimal length of time that a patient should remain on PD in order to avoid the occurrence of this complication [7]. The lack of well-defined prognostic criteria limits the opportunity to detect patients being at high-risk for developing EPS; thus, the diagnosis is often late, when the patients develop ultrafiltration failure and gastro-intestinal complications [1, 7]. The current standard-of-care in the management of EPS is relied on discontinuation of PD, transition to hemodialysis and nutritional support of patients [1, 7]. The potential benefit of corticosteroids, tamoxifen or other immunosuppressive agents that have been used over the years in pharmacological treatment of EPS remains unclear in the absence of clinical-trial evidence to prove their efficacy and safety [8–10]. EPS is sometimes treated with surgical enterolysis [11, 12]; however, this surgical procedure is complex and is rarely performed in the absence of definite supportive data and due to the limited clinical experience [1].

Herein, we report the clinical course of a 28-year-old patient who developed a severe form of calcifying EPS that was successfully managed with prolonged administration of tamoxifen. Over a 10-year-long follow-up, the patient experienced minimal gastrointestinal complications and maintained an optimal health-related quality of life without manifesting any tamoxifen-inducible adverse events.

## Case presentation

A 28-year-old Caucasian male with end-stage-renal-disease due to juvenile nephronophthisis developed a severe form of calcifying EPS approximately 10 years ago. His medical history included the presence of hypertension and mitral valve prolapsing without significant regurgitation. The patient was started on continuous ambulatory PD therapy at the age of 13 years and his initial PD regimen included 4 dwells of dialysate glucose solutions 1.36% with fill volume 2 L. Since the peritoneal equilibrium tests showed persistently a high transport status (dialysate-to-peritoneal creatinine ratio: 0.78), 2 years later the patient was switched to continuous cyclic PD with 4 night-time dwells of dialysate glucose solutions 1.36% (duration: 8 h; cycles: 4; fill volume: 2 L) and a daytime dwell with icodextrin (fill volume: 1.5 L). The patient followed a typical clinical course characterized by adequately delivered dialysis and sufficient peritoneal ultrafiltration of ~ 1.5 L/day with a progressive intensification of his PD regimen (continuous cyclic PD with 4 nighttime dwells of dialysate glucose solutions 2.36% and a daytime dwell with icodextrin) till the age of 19 years. Over this period, the patient had secondary hyperparathyroidism and received therapy with calcium-containing phosphate binders and active vitamin D analogs because the patient could not tolerate oral administration of cinacalcet due to gastro-intestinal side effects. The only PD-related complications were 3 episodes of peritonitis due to *Staph. Epidermidis* successfully treated with intraperitoneal antibiotics.

**Fig. 1:**
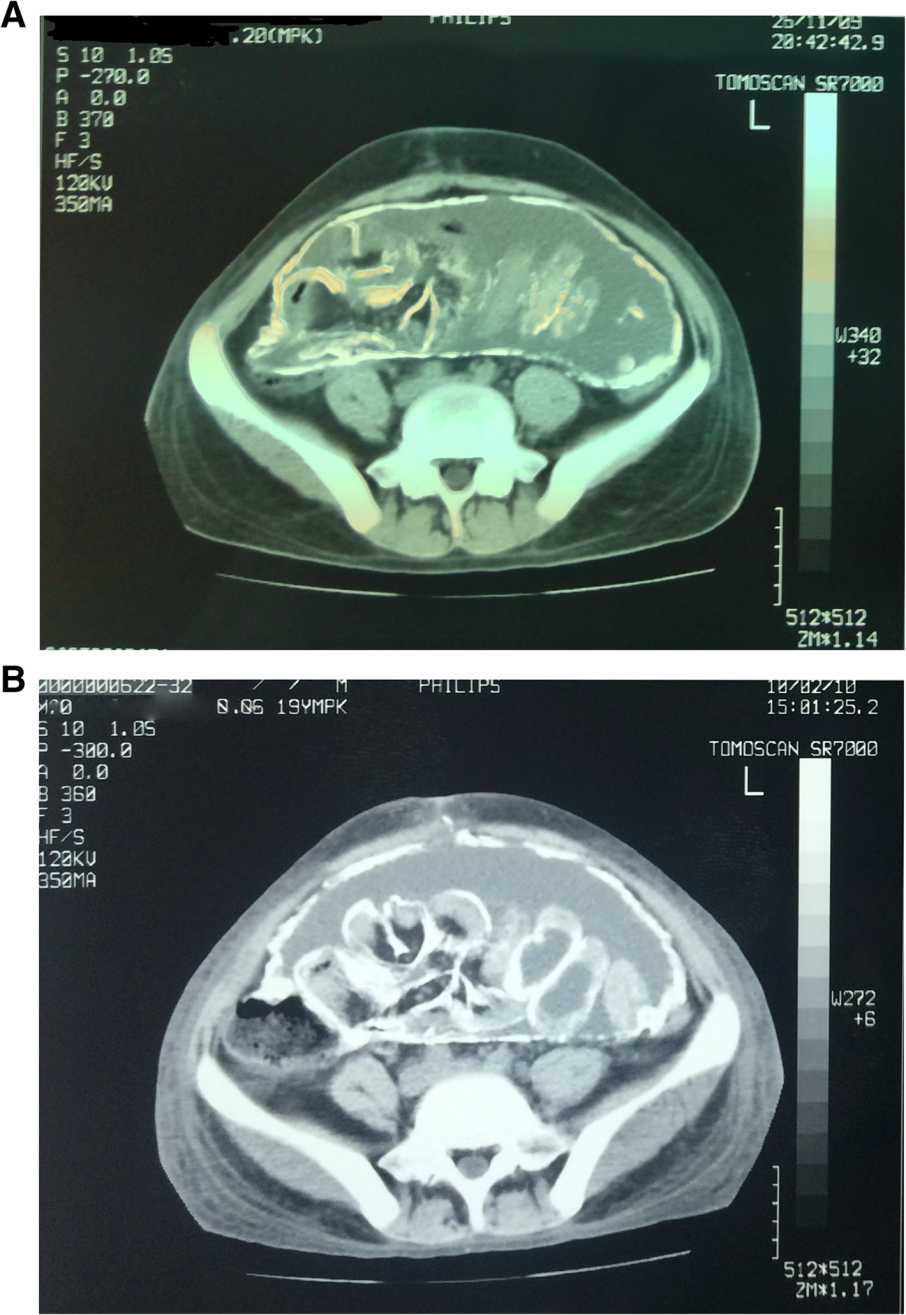
**a** Abdominal CT scan (Nov 2009) showing excessive peritoneal thickening, multiple intra-abdominal adhesions of bowel loops and a calcified fibrous cocoon wrapped around the bowel; **b** abdominal CT scan (Feb 2010) showing persistence of calcified intra-abdominal lesions and peritoneal thickening

After 6 years on automated PD, the patient developed a progressive reduction in peritoneal ultrafiltration that necessitated the intensification of the PD regimen. In parallel, the patient reported symptoms of atypical abdominal pain, anorexia, vomiting and constipation, raising the clinical suspicion of EPS. A computed-tomography (CT) scan in November 2009 showed a calcified fibrous cocoon wrapped around the bowel, extensive peritoneal thickening and intra-abdominal adhesions of calcified intestinal loops (Fig. 1a), confirming the diagnosis of calcifying EPS. The initial therapeutic approach included the removal of the peritoneal catheter, transfer of the patient to hemodialysis and administration of corticosteroids (prednisone at a dose of 40 mg/day) in combination with tamoxifen 20 mg/day. The clinical course of the patient over the subsequent 3 months was dramatic due to severe symptoms of total bowel obstruction (ileus stage), malnutrition and rapid weight loss of 20 kg that mandated the prolonged in-hospital treatment and nutritional support with parenteral supplements (1 lt Oliclinomel N4/24-h during the first month and 1lt Oliclinomel N4 3 times per week during dialysis thereafter till the patient was given discharge from the hospital).

**Fig. 2:**
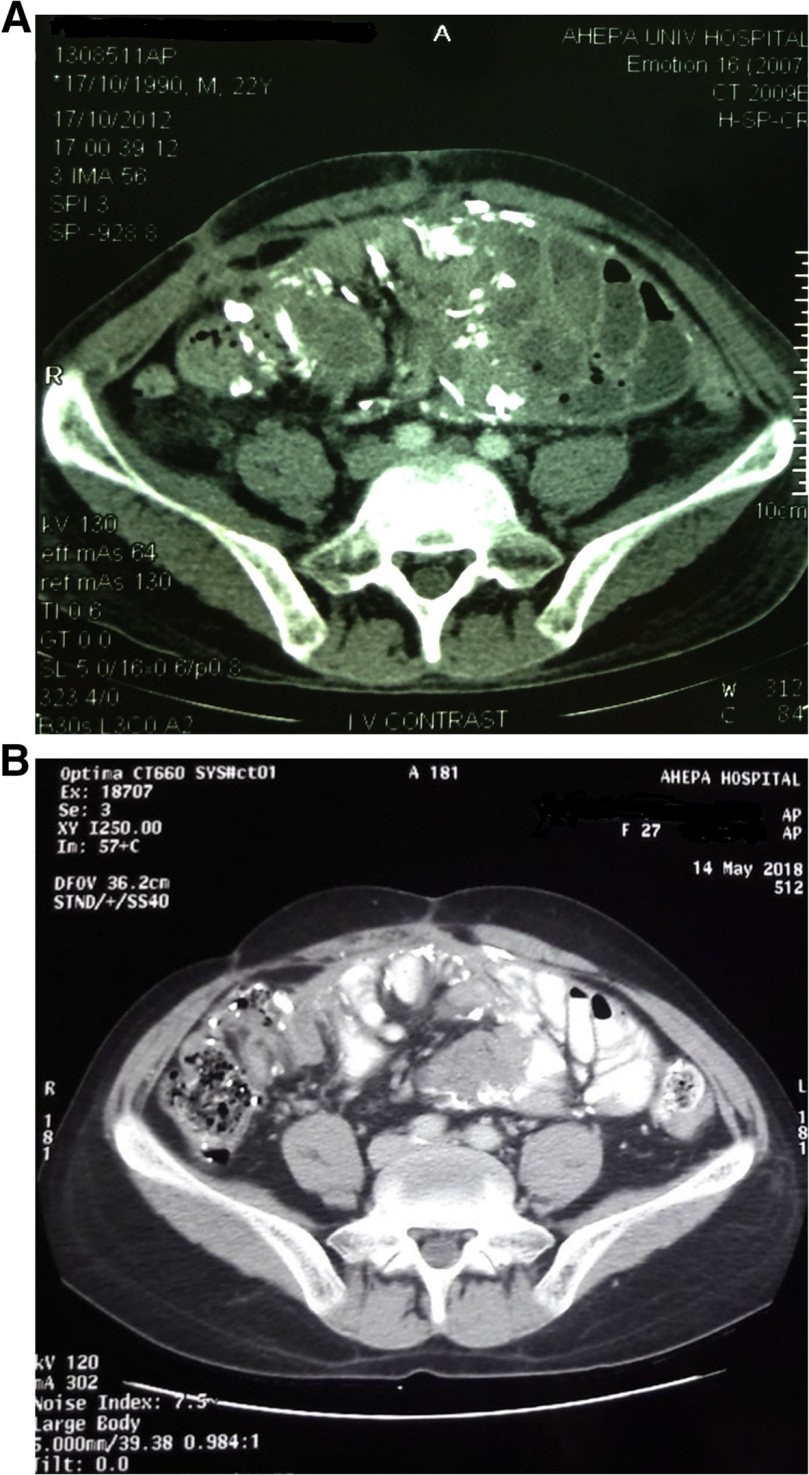
**a** Abdominal CT scan after the discontinuation of steroids (Oct 2012) showing a similar radiological picture in comparison with earlier CT scans; b abdominal CT scan (May 2018) showing minimal improvement of the radiological picture over a 10-year-long follow-up. The high density in (**b**) is due to oral intake of a water-soluble contrast agent for the abdominal CT scan evaluation

Despite the severe initial clinical presentation, the patient followed a satisfactory clinical course over the next 6 months with remission of gastrointestinal complications and progressive improvement in his nutritional status (Table 1). Despite the clinical improvement, repeated CT scans showed the persistence of peritoneal thickening and intra-abdominal calcifications with modest improvement in radiological picture over time (Figs. 1 and 2). In this context, prednisone was gradually tapered over 6 months to 10 mg/day and was permanently discontinued after 18 months of therapy. Tamoxifen remained unmodified and was administered at a low-dose of 20 mg/day over a 10-year-long period. Long-term therapy with tamoxifen was well-tolerated and was not accompanied by any thrombo-embolic complications or other drug-related adverse effects. During follow-up, the patient experienced minimal gastrointestinal complications, maintained a stable nutritional status and required only 7 short-term hospitalizations due to episodes of incomplete bowel obstruction. All these episodes were mild and were successfully managed with conservative treatment (i.e., gastro-intestinal rest, antibiotics, etch). Immediate surgical treatment, corticosteroids or parenteric nutritional support was never required during follow-up. As shown in Table 1, the management of secondary hyperparathyroidism over this period was based on low-to-moderate doses of intravenously administered paricalcitol as well as on optimal oral treatment with phosphate-binders.

**Table 1:** Haematological and biochemical profile of the patient at disease onset and during the 10-year-long follow-up

Parameter	Dec 2009	Feb 2010	June 2010	Jan 2012	Jan 2013	Jan 2015	Jan 2017	Jan 2019
Body weight (kg)	78	58	63	67	78	73	71	70
Hemoglobin (g/dl)	8.9	10.4	11.8	11.2	12.1	12.0	12.2	11.9
Serum urea (mg/dl)	78	91	150	176	168	128	145	133
Serum creatinine (mg/dl)	8.8	9.4	10.7	11.3	11.2	11.8	12.0	12.5
Serum albumin (g/dl)	2.4	3.3	3.8	3.7	4.1	4.0	4.2	4.1
Serum calcium (mg/dl)	7.5	8.3	8.9	8.4	8.7	9.0	9.3	9.2
Serum phosphate (mg/dl)	2.6	3.5	4.5	6.5	5.5	4.9	5.8	5.5
PTH (pmol/l)	22.9	24.9	29.4	49.5	51.0	54.2	43.0	29.6
C-reactive-protein (mg/dl)	10.8	7.1	2.1	1.3	1.23	1.1	1.0	1.3
Treatment for the secondary hyperparathyroidism								
Calcium carbonate	–	–	–	1500 mg/d	1500 mg/d	1500 mg/d	1500 mg/d	3000 mg/d
Paricalcitol	–	–	2.5 μgr/HD	7.5 μgr/HD	5.0 μgr/HD	5.0 μgr/HD	5.0 μgr/HD	7.5 μgr/HD

## Discussion and conclusions

This report highlights a severe case of calcifying EPS in a PD patient who was successfully managed with conservative treatment and prolonged administration of tamoxifen. Despite the fact that the mortality rate of severe EPS is reported to be ~ 25% within the first year of diagnosis [13, 14], the patient in our case-report followed a 10-year-long course with a clinically meaningful improvement in gastrointestinal complications, nutritional status and in the overall health-related quality of life. Although the observational nature of our data cannot demonstrate direct cause-and-effect associations, our case-report provides evidence that support the safety and efficacy of long-term therapy with low-dose tamoxifen in patients with severe calcifying EPS.

Tamoxifen is a non-steroidal anti-estrogen that has been used in the treatment of several fibrotic diseases, such as retro-peritoneal fibrosis and EPS [1]. Although the exact mechanism of action is not yet fully clear, experimental studies suggest that the anti-fibrotic properties of tamoxifen are mediated through estrogen receptor (ER)-independent pathways [15]. In this direction, in-vitro studies using human peritoneal mesothelial cells have shown that tamoxifen down-regulates the gene expression of connective-tissue-growth-factor and inhibits the collagen synthesis mediated through the transforming-growth-factor-β (TGF-β) pathway [16, 17]. In animal models of PD, administration of tamoxifen is shown to ameliorate the progression of peritoneal thickening and improve peritoneal membrane function through inhibition of the process of mesothelial-to-mesenchymal transition [18].

The first clinical report on successful use of tamoxifen in the treatment of PD-related EPS was published by Allaria et al. in 1999 [19]. Since then, the potential benefit of this agent is supported by a number of case-reports or case-series showing reduced inflammation and improvement in intestinal function in response to tamoxifen therapy [6, 9, 20–23]. In the Dutch multi-centre EPS study [22], the survival status of 24 tamoxifen-treated patients with EPS was retrospectively compared with that of 39 controls with EPS who did not receive tamoxifen therapy. In Cox-regression analysis adjusted for age, gender, concomitant use of corticosteroids and other confounding factors, the use of tamoxifen was associated with a marginally non-significant survival benefit (Hazard Ratio: 0.39, *P* = 0.056) [22]. In a subsequent series of 14 EPS-prone patients, identified on the basis of presence of at least 2 documented risk factors for developing EPS, it was shown that over a median follow-up of 54.05 months, administration of tamoxifen alone or in combination with corticosteroids was associated with lower risk of progression to clinically overt EPS [20].

The present case-report confirms and expands the findings of the above case-series providing rationale for the safe and effective long-term use of low-dose tamoxifen in severe cases of EPS. Although the accumulated clinical experience until now is suggestive of a benefit with an initial course of tamoxifen therapy for 6–12 months, the optimal duration of therapy and the clinical or radiological criteria to guide the pharmacological treatment of EPS remain unspecified. In the patient of our case-report, introduction of tamoxifen in combination with corticosteroids was associated with improvement in gastrointestinal complications and nutritional status over the first 6–12 months, whereas the radiological lesions of calcifying EPS persisted in repeated abdominal CT scans during follow-up. Taking into consideration the clinical response as well as the severity of radiological picture, we maintained low-dose tamoxifen therapy for a long period. The development and persistence of calcifying peritoneal lesions is possibly related to the severe secondary hyperparathyroidism of the patient and his background therapy with calcium-containing phosphate binders and active vitamin D analogs (Table 1). The rationale for the prolonged administration of tamoxifen was that this agent would mediate long-term anti-fibrotic and/or anti-inflammatory actions, without expecting regression of severe calcifying lesions in response to tamoxifen therapy. In our opinion, corticosteroids was the component of therapy that possibly down-regulated the inflammatory process during the initial phase (i.e., when the patient was at an ileus stage), whereas administration of tamoxifen was presumably more important in maintaining long-term remission through anti-fibrotic/anti-proliferative actions. It has to be noted, however, that long-term administration of tamoxifen requires careful monitoring of patients for the identification of drug-related adverse effects. Tamoxifen use has been associated with higher risk of thromboembolic events, flushes, endometrial carcinoma, thrombopenia and calciphylaxis [1, 24]. Whether the clinical course of our patient would be similarly successful without exerting the patient to potential risks arising from the long-term tamoxifen use remains a question that can not be addressed by our case report. The efficacy and safety profile of this agent warrants further investigation in larger case-series or in randomized controlled trials.

In conclusion, this case-report provides some evidence that support the long-term safety and efficacy of low-dose tamoxifen in severe cases of calcifying EPS. Since the rarity of EPS is a barrier against the design of a randomized controlled trial, evidence derived from case-reports and case-series remain an important step in order to quantify the benefit/risk ratio of tamoxifen and other therapies. Individualization on the basis of the disease severity, response to therapy and occurrence of adverse events is, perhaps, the optimal therapeutic approach of this rare, but life-threatening PD-related complication.

## Data Availability

All data collected from this patient were obtained from the AHEPA University Hospital of Thessaloniki and and are available in this paper.

## Abbreviations

CT: Computed tomography
EPS: Encapsulating peritoneal sclerosis
PD: Peritoneal dialysis
TGF-β: Transforming-growth-factor-β

## References

[1] Korte MR, Sampimon DE, Betjes MG, Krediet RT (2011). Encapsulating peritoneal sclerosis: the state of affairs. Nat Rev Nephrol.

[2] Brown MC, Simpson K, Kerssens JJ, Mactier RA (2009). Encapsulating peritoneal sclerosis in the new millennium: a national cohort study. Clin J Am Soc Nephrol.

[3] Johnson DW, Cho Y, Livingston BE (2010). Encapsulating peritoneal sclerosis: incidence, predictors, and outcomes. Kidney Int.

[4] Tseng CC, Chen JB, Wang IK (2018). Incidence and outcomes of encapsulating peritoneal sclerosis (EPS) and factors associated with severe EPS. PLoS One.

[5] Lambie MR, Chess J, Summers AM, Williams PF, Topley N, Davies SJ (2016). Peritoneal inflammation precedes encapsulating peritoneal sclerosis: results from the GLOBAL fluid study. Nephrol Dial Transplant.

[6] Balasubramaniam G, Brown EA, Davenport A (2009). The pan-Thames EPS study: treatment and outcomes of encapsulating peritoneal sclerosis. Nephrol Dial Transplant.

[7] Brown EA, Bargman J, van Biesen W (2017). Length of time on peritoneal Dialysis and encapsulating peritoneal sclerosis - position paper for ISPD: 2017 update. Perit Dial Int.

[8] Fagugli RM, Selvi A, Quintaliani G, Bianchi M, Buoncristiani U (1999). Immunosuppressive treatment for sclerosing peritonitis. Nephrol Dial Transplant.

[9] Lafrance JP, Letourneau I, Ouimet D (2008). Successful treatment of encapsulating peritoneal sclerosis with immunosuppressive therapy. Am J Kidney Dis.

[10] Wong CF, Beshir S, Khalil A, Pai P, Ahmad R (2005). Successful treatment of encapsulating peritoneal sclerosis with azathioprine and prednisolone. Perit Dial Int.

[11] Kawanishi H, Watanabe H, Moriishi M, Tsuchiya S (2005). Successful surgical management of encapsulating peritoneal sclerosis. Perit Dial Int.

[12] Kawanishi H, Moriishi M, Tsuchiya S (2006). Experience of 100 surgical cases of encapsulating peritoneal sclerosis: investigation of recurrent cases after surgery. Adv Perit Dial.

[13] Latus J, Ulmer C, Fritz P (2013). Encapsulating peritoneal sclerosis: a rare, serious but potentially curable complication of peritoneal dialysis-experience of a referral Centre in Germany. Nephrol Dial Transplant.

[14] Ulmer C, Braun N, Rieber F (2013). Efficacy and morbidity of surgical therapy in late-stage encapsulating peritoneal sclerosis. Surgery..

[15] Braun N, Fritz P, Biegger D (2011). Difference in the expression of hormone receptors and fibrotic markers in the human peritoneum--implications for therapeutic targets to prevent encapsulating peritoneal sclerosis. Perit Dial Int.

[16] Huang JW, Yen CJ, Wu HY (2011). Tamoxifen downregulates connective tissue growth factor to ameliorate peritoneal fibrosis. Blood Purif.

[17] Yan P, Tang H, Chen X, et al. Tamoxifen attenuates dialysate-induced peritoneal fibrosis by inhibiting GSK-3beta/beta-catenin axis activation. Biosci Rep. 2018;38(6). 10.1042/BSR20180240. 10.1042/BSR20180240 PMC6246765 30061174

[18] Loureiro J, Sandoval P, del Peso G (2013). Tamoxifen ameliorates peritoneal membrane damage by blocking mesothelial to mesenchymal transition in peritoneal dialysis. PLoS One.

[19] Allaria PM, Giangrande A, Gandini E, Pisoni IB (1999). Continuous ambulatory peritoneal dialysis and sclerosing encapsulating peritonitis: tamoxifen as a new therapeutic agent?. J Nephrol.

[20] De Sousa-Amorim E, del Peso G, Bajo MA (2014). Can EPS development be avoided with early interventions? The potential role of tamoxifen--a single-center study. Perit Dial Int.

[21] Eltoum MA, Wright S, Atchley J, Mason JC (2006). Four consecutive cases of peritoneal dialysis-related encapsulating peritoneal sclerosis treated successfully with tamoxifen. Perit Dial Int.

[22] Korte MR, Fieren MW, Sampimon DE, Lingsma HF, Weimar W, Betjes MG (2011). Tamoxifen is associated with lower mortality of encapsulating peritoneal sclerosis: results of the Dutch multicentre EPS study. Nephrol Dial Transplant.

[23] Poultsidi A, Liakopoulos V, Eleftheriadis T, Zarogiannis S, Bouchlariotou S, Stefanidis I (2006). Gross calcification of the small bowel in a continuous ambulatory peritoneal dialysis patient with sclerosing peritonitis. Adv Perit Dial.

[24] Fisher B, Costantino JP, Wickerham DL (2005). Tamoxifen for the prevention of breast cancer: current status of the National Surgical Adjuvant Breast and bowel project P-1 study. J Natl Cancer Inst.

